# Exploring mental health implications of informal caregiving for the older adult within the Hispanic community: an in-depth cross-cultural analysis of depression symptoms

**DOI:** 10.3389/fpubh.2025.1610733

**Published:** 2025-08-26

**Authors:** Angel Muñoz-Alicea, William Suarez-Gomez

**Affiliations:** ^1^Department of Psychology, College of Behavioral Sciences and Community Affairs, Pontifical Catholic University of Puerto Rico, Ponce, Puerto Rico; ^2^Health Equity, Administration & Technology Department, School of Health Science, Health Services and Nursing, Lehman College, City University of New York, Bronx, NY, United States

**Keywords:** informal caregivers, older adults, Hispanic, cross-cultural design, depression, spirituality, familism

## Abstract

This study contributes to a deeper understanding of depression among Hispanic informal caregivers of older adults. As aging populations grow across Latin America and the U.S., it is essential to examine how sociocultural and demographic variables influence caregivers' mental health. A non-experimental, cross-cultural design quantitative research was conducted in Colombia, Mexico, and Puerto Rico-countries with strong representation in the U.S. Using the Psych Data platform and the Patient Health Questionnaire-9 (PHQ-9), a total of 1,194 informal caregivers were assessed for self-rated depressive symptoms and perceived social support. Multivariate statistical analyses were conducted using linear regression analyses and *t*-test to investigate the correlations between the sociodemographic profiles and depression symptoms of caregivers for older adults in primary care by country. The findings highlight associations between informal caregiving and gender, depression, age, education, employment, spirituality, and familism. Results showed that employment was positively associated with depressive symptoms (*B* = 1.452, *p* < 0.01), suggesting that work may add strain rather than serve as a buffer. Higher educational attainment appeared protective, potentially by improving access to mental health resources and coping strategies. Unexpectedly, spirituality also emerged as a significant predictor of increased depressive symptoms, challenging previous research that frames spirituality as protective. Considering the escalating population of minority older adults facing health decline, it is necessary to enhance comprehension of how these multidimensional variables influence caregiving. These findings underscore the need to tailor ethnocultural interventions to address caregiver burden and mental health disparities in underserved populations.

## Introduction

The United States and several Caribbean and Latin American nations are facing growing strain on their national budgets and health services due to the increasing demand for long-term support for their aging populations (1–4). In the United States (US), Mexico, Puerto Rico, and Colombia, the population age 65 and over is 18, 8.6, 23.4, and 9.4%, respectively (5, 6), and many of them prefer to remain at home for as long as possible. Empowering the older adult to remain in their homes promotes active lifestyles and preserves their independence, but it may also require family support and a culturally competent approach (7, 8). Many people will take on the role of a caregiver at some point in their lives. By gaining a better understanding of the factors involved in family caregiving and the effects on caregivers, we can improve support initiatives and promote greater equity (9). Family and/or informal caregivers for the older adult remain an underrecognized yet essential support system for individuals with cognitive impairments or functional limitations who remain at home. However, unpaid informal family caregivers serve as the primary support system for long-term care within individuals' residences, particularly among middle-aged and older adult individuals who provide care for their family members (10). An estimated one in five citizens in the US (1), one in six in Mexico (11) and Puerto Rico (12, 13), and one in 10 citizens in Colombia (14) have assumed a caregiver's role yearly. The purpose of this non-experimental, cross-cultural study is to enhance comprehension of multidimensional factors that impact depression symptoms among caregivers for the elderly in three Hispanic communities linked to migrants in the US.

Caregiving, when viewed from an anthropological perspective, is shaped by cultural constructs within society and influenced by biological, historical, and other macro and microsocial factors (15). Caregivers' motivations and willingness to care are underpinned by multidimensional factors such as religious and philosophical beliefs, a sense of duty, and the desire to reciprocate play significant roles in shaping the bonds, values, and beliefs associated with caregiving (2, 15, 16). For instance, the principle of honoring your parent rooted in Jewish-Christian-Catholic values, has been a foundational aspect of Hispanic families, reflected in some legal codes and transmitted across generations within the wider society.

However, the individual in the caregiving role focuses on immediate tasks, guided by representations that validate both the successes and failures of caregiving in a social context (15). As a result, most of the family caregivers face an elevated risk of burn-out, loneliness, anxiety, and depression due to the stress inherent in caring for a loved one (15–17). This risk may vary based on a range of factors.

In this context, this study investigates the relationship between the sociodemographic characteristics of informal caregivers and their levels of depressive symptoms, examines significant differences, and analyzes the extent to which psychosocial factors predict depressive symptoms in a sample from three Hispanic countries: Colombia, Mexico and Puerto Rico. The study provides additional insight into the impact of care-related depression among Hispanic caregivers in their country of origin. However, it is essential to recognize that how individuals address and cope with depression may differ significantly across diverse cultural contexts (15–18).

This study was guided by two complementary theoretical models. The first, the family ecological structural-systems model, conceptualizes caregiving across three interconnected levels: the microsystem (caregiver and immediate family), the mesosystem (community interactions and support), and the macrosystem (structural conditions influencing caregiving). In parallel, also guided by the model proposed by Falzarano et al. (19) emphasizes the role of familism and social support in shaping caregiver interventions, underscoring the need for culturally responsive strategies that address the specific needs of diverse ethnic groups.

## Literature review

In the US literature, studies on family caregiver interventions have predominantly included participants who are white, non-Hispanic, and highly educated. This demographic restricts the applicability of the findings to a broader population. Hispanics in the US, in general, have had the highest life expectancy at birth of all groups (81.8 years), despite facing a disadvantaged socioeconomic profile (20–22). Among Hispanic migrant subgroups in the US, Puerto Ricans exhibit the highest mortality rate at 605.7 deaths per 100,000 individuals, followed by Mexicans at 523.7, Cubans at 489.1, and Colombians at 390 (23). Notably, all these rates are lower than those of non-Hispanic Caucasians, who have a mortality rate of 784, and non-Hispanic Black people, whose rate stands at 924 (24). These rates highlight the need to explore alternative methods of care and the challenge of understanding the macro- and micro sociocultural factors that contribute to serving these communities.

### Cultural norms and caregiving

The personal ethnocultural and life experiences shape caregivers' perceptions, actions, and social approaches. For instance, in Hispanic cultures, familism often is linked to specific gender roles and those who do not fully embrace these values may experience higher levels of depression and anxiety (19, 25). Men's roles are typically seen as providers, protectors, emotionally controlled, and strength, while caregiving is often viewed as less relevant to their roles (26, 27). However, the data from Latino/Hispanic caregivers in the US discussed by Whitney et al. (28) identified them as male, high school diploma, being from Generation X, and caring for a parent or stepparent. The Hispanic women's role, in contrast, is expected to be as the nurturer, “*the self-sacrificing mother*” and the moral anchors within the family, which is described as Marianism (25, 29). Matriarchal structures also play a significant role, with mothers and grandmothers providing essential emotional support. Although traditionally assigned to women, caregivers must navigate the challenges of social expectations. Moreover, caregivers often appear to be divinely chosen, with God serving as a source of strength during challenging times when resilience is needed to overcome adversity (27, 30).

General studies of Latin America estimate that 37% of men and 63% of women spend over 60% of their working hours on unpaid care activities for infants and older adults (31). In Mexico, for instance, women account for 71% of care hours for individuals over 60 (32) whilst in Colombia feminization and social segregation are the main two adjective of their older adult population (33). In Puerto Rico, caregiving patterns are similar, with women over 65 often facing greater vulnerability due to limited participation in the formal labor market and economic dependency. Nevertheless, it can be asserted that within these societies, the role of caregiving is developed through the ongoing observation of the caregiving behaviors exhibited by close relatives.

Family plays a pivotal role in caregiving. Among the US white-non-Hispanic population, caregivers often identify themselves as “spouses/partners, children, siblings, or friends” of the care recipient rather than simply as “caregivers.” In some instances, within the communities of non-Hispanic Black people and Hispanic people, it is not uncommon for extended family members to take on caregiving roles. Familism, which is more common among non-Hispanic Black people and Hispanic communities, is a positive cultural value that highlights the importance of collectivism and close-knit family relationships (19, 34). McCleary and Blain (35) have shown that individuals with more traditional beliefs may assume their caregiving role out of obligation, which can exacerbate feelings of role captivity and distress. Tran et al. (36) posit that ethnocultural values may impact the perceived caregiving experience, burdens, changes in relationships with care recipients, and support. It is understood that familism varies based on the caregiver's relationship to the care recipient and its impact on psychosocial functioning (37). However, while *familism* can provide a robust informal support system, paradoxically may contribute to caregiver burden by creating strong obligations and deterring the utilization of external resources (19).

Self-efficacy, regarded as a cultural value, may be construed as originating from internalized expectations and beliefs (16). For instance, “personalization” among Latino caregiving dynamics may relate to genuine rapport, trustful bounding, which in generally is linked to reservations about including non-family members in care, respect for elders, and a collectivist approach (38, 39). While these values promote belonging and interdependence, they can also create stereotypes and stigmas around mental health.

Racial and ethnic variables play a role in shaping individuals' perceptions and evaluations of contextual elements. These factors, in turn, exert diverse influences on stress and coping mechanisms, with eventual impacts on caregiver outcomes be they positive and/or negative (19, 36). Peer-reviewed publications on caregivers' profiles are relatively scarce, and even more uncommon are cross-cultural studies contrasting Hispanic family caregivers' profiles. A range of valuable publications, predominantly in English, has explored this topic through a “one-size-fits-all” lens (39–42). Yet, cultural norms surrounding family obligation, familism, and ethnicity influence motivations for informal care, although the underlying factors remain unclear (16, 17, 29).

While it is recognized that current discussions and immigration policies in the US may have reinforced some stereotypes about the Hispanic community, it is important to acknowledge the rising population of ethnocultural minority older adults who are experiencing health challenges (43). This underscores the necessity for a deeper understanding of how cultural factors can affect and enhance caregiving. In the US, Mexicans, Puerto Ricans, and Colombians are among the top five largest populations in the Hispanic diaspora, but their cultural background and migratory status are not the same. In general, the literature is limited in exploring how individual and collective identity issues, along with associated emotions, influence the distribution of caregiving tasks. It overlooks the complexity of care decisions and their relationship to feelings and behaviors that are regarded as “appropriate” according to social norms of the nation (44). However, the literature also highlights that culturally adapted or linguistically congruent tools facilitate a better understanding of a phenomenon and may ultimately lead to better strategies for educating, training, or prompting groups in need, thus enhancing their skills to address their own challenges (45–47).

### Psychological stressors to shape

Numerous studies indicate that caregivers frequently experience poorer health outcomes compared to non-caregivers (48). The caregiver's dedication and bond with the older adult lead to a burden of suffering from the adult's deterioration and their own emotional pain (49). Based on the AARP report “Caregiving in the United States” 29% of family members involved in high-intensity caregiving reported experiencing significant physical strain, while 49% reported notable emotional strain (40). Older adults with notable prevalence of depression, ranging from 5 to 30%, have significant effects, increasing the caregiver's burden (50). During the COVID-19 pandemic, Hurtado-Vega (51) noted that nearly 40% of Mexicans over the age of 60 with dependency conditions received care from informal caregivers. Precisely, Carrillo-Cervantes et al. indicate that Mexican caregivers experienced an additional impact on their own mental health, predominantly facing anxiety (27%) and a clinical concern with depression (14.9%), while a significant number (66.2%) experienced profound loneliness. These authors also found that psychosocial factors, the ages of caregivers and care recipients, and the duration of care accounted for 36% of the variance in role adoption, with loneliness identified as a significant predictor (52).

Alonso-Rodriguez et al. found that Colombian caregivers often experience anxiety due to the extensive responsibilities and exhaustion associated with caregiving. They noted that this anxiety is also connected to a lack of skills and limited access to resources needed to effectively manage these challenges (53). However, once caregivers receive training, they can develop effective decision-making skills and find meaning in their experiences. Training helps them cultivate coping strategies that reduce their anxiety levels (45). Increased knowledge and skills in caregiving enable caregivers to manage adversity more effectively and adopt active coping strategies, leading to reduced stress (54).

### The interplay of sociocultural factors

Family systems, race, gender, income, religious affiliation, and culture may raise challenges when there is significant interdependence among the care recipient, live-in caregiver, and family support, as each relies on the others for their wellbeing (15, 48, 55–58). Family caregivers encounter ethical dilemmas arising from conflicting loyalties to the older care recipient and their own wellbeing (59). Caregivers may experience significant guilt or shame when expressing negative feelings or concerns about caring for the elderly (15, 16, 29). However, communities that are more socially cohesive may have greater trust in helping one another, which could be influenced by the type of living area—rural vs. urban—as well as local traditions (60). Ignoring these factors may harm the relationship between the caregiver and the care recipient, leading to less favorable outcomes (16).

Social support may reduce burden and depression, while warm and supportive family relationships predict positive caregiving aspects. Research indicates that certain caregivers may experience higher levels of burden compared to others. For instance, those in roles with more intensive care responsibilities, including managing challenging behaviors in addition to assisting with daily activities, may experience heightened levels of burden and depression (19). The literature indicates that the health outcomes of caregivers are significantly influenced by race and gender (61). The age of the caregiver is also linked to their feeling of loneliness, which may correlate with depression (62). Schulz et al. (4) highlighted that the caregiving intensity (defined by the number of hours dedicated to caregiving per week or month), family ties (particularly among wives), and cohabitation with the care recipient serve as relatively robust indicators of negative psychological repercussions. Kellner et al. (63) found that social embarrassment significantly influences caregiver depression, with consistent effects across different cultural groups (64). These variables emphasize the critical importance of addressing disparities to ensure equitable care for all.

Another critical aspect to consider is that many older adults prefer to receive care from family members. This trend has resulted in increased responsibilities for family caregivers and presents significant challenges for healthcare institutions. To understand the differences in perceptions, we focused on exploring the caregiving role embedded in the cultural roots of their country of birth. Considering the escalating ethnocultural population of minority older adults facing health decline, it is necessary to enhance comprehension of how cultural factors impact the caregiving process.

## Materials and methods

This study employed a quantitative methodology with an exploratory, descriptive-correlational design, and was conducted in a sample from three Hispanic countries linked to migrants in the US. Specifically, the research was conducted as a non-experimental, cross-sectional study, meaning the variables were not manipulated and were measured at a single point in time.

During the sample selection phase, no official registry or database was available to determine the total number of informal caregivers in the participating countries. Consequently, calculating a statistically representative sample size was not feasible. Due to the challenges in accessing informal caregivers—particularly related to time constraints—a non-probability, convenience sampling method was used. This approach involves selecting participants based on predefined criteria and availability, and although it is commonly used in exploratory research, it limits the generalizability of findings to the broader population. The participants were recruited through collaborative efforts involving four academic institutions.

To be eligible for this study, caregivers had to be caring for someone who was not institutionalized. In addition, they had to meet inclusion criteria such as: being of legal age in their country of residence, providing care or support to at least one person aged 60 or older, not receiving a salary for their caregiving duties, being literate and able to use an electronic device (computer, tablet, or smartphone), and voluntarily agreeing to participate. Exclusion criteria included the presence of severe mental health conditions that could impair the participant's ability to understand or complete the study.

### Participants

This research study included a total of 1,194 caregivers for older-adult individuals from Mexico (51%), Puerto Rico (32.3%), and Colombia (16.7%). Caregivers were identified as a family member—either a spouse, adult child, sibling, in-law, cousin, nephew or niece or close friend—who provided most of the day–to–day care for the cognitively or physically impaired individual. A variety of methods were employed to recruit participants for the study. Family caregivers of older adult individuals were invited and/or contacted through support groups, parishes or churches, care centers and/or daytime (respire) programs. In addition, in some areas included in the study, the community leaders were invited to disseminate the promotional poster. The invitations to participate were distributed in the municipalities or communities included, also using telephone messaging (SMS), WhatsApp groups, and/or contacts. Although the study was not experimental in nature, the sample distribution from Puerto Rico and Mexico effectively included participants from almost every region of their respective national territories, providing a comprehensive overview of the populations. Individuals from 28 out of the 32 states of the United States of Mexico participated actively and, in Puerto Rico, residents from all the 78 municipalities participated. In contrast, the sample distribution in Colombia was more limited. Surveyors focused on only 16 out of the country's 32 administrative geographic divisions, which may not fully reflect the Colombia's diversity and various demographic groups.

### Ethical considerations

Ethical approval for this study was obtained from the Institutional Review Board (IRB) in Puerto Rico and from the Research Ethics Committee in Mexico; in Colombia, ethical approval was not required. All participants provided informed consent before participating in the survey, which was conducted electronically, using the digital platform PsychData (65). To uphold privacy, security, and confidentiality, the data was devoid of any information that could potentially identify the participants, and IP addresses were not collected.

### Secondary data and scope of analysis

This study is a secondary analysis of data originally collected for a previous project (66). The current research builds on the original findings by narrowing the focus to the psychosocial factors such as educational level, employment, spirituality, and social support as protective factors against depressive symptomatology in informal caregivers. Thus, offering new insights and interpretations not addressed in the earlier publication. Only participants who completed the full Patient Health Questionnaire (PHQ-9) were included in this analysis, thereby increasing the sample size.

### Questionnaires

The study consisted of a four-part self-assessment digital questionnaire with an estimated completion time of 20 min (65). The first part was an initial demographic assessment about the cognitively or physically impaired older adult person (caretaker) based on a standardized set of questions, to identify the needs and level of dependency on the primary informal/family caregiver. This part of the instrument also included items that build the caregivers' profile by country, including gender, age, marital status, number of children, employment, income, etc. Their situation and the relationship to the care recipient were assessed. The remaining sections of the instrument included a structured and standardized set of items designed to assess symptoms of mental disorders, specifically using the Patient Health Questionnaire-9 (PHQ-9) to evaluate depressive symptoms. Caregivers were asked to self-assess and to rate their health status. In this paper, we focus on signs of depression in the primary informal caregiver.

### Tools and validation

The research was carried out in Spanish, with careful attention to idiomatic expressions and cultural differences that could impact the validity of intergroup comparisons. Given that, in some cases, a verbatim reproduction that precisely mirrors the original document, even in groups with the same main language, may not be the most culturally suitable method (67). Prior to distribution, the instruments were piloted with focus groups comprised of students in Mexico and Colombia. This pilot study sought to evaluate the clarity of the questionnaire, ensuring that the questions were precisely defined and easily understandable by the population. It is important to acknowledge that the participants in the focus groups had some differences compared to the characteristics of the intended study sample.

### Patient health questionnaire-9 (PHQ-9)

The official Spanish version used of the Patient Health Questionnaire-9 (PHQ-9), was translated and validated by Pfizer Inc. This culturally and linguistically adapted version has been widely employed and psychometrically validated in clinical and community settings across Spanish-speaking populations, including Colombia, Mexico, and Puerto Rico. Across these contexts, the PHQ-9 has consistently demonstrated strong internal consistency, with Cronbach's alpha coefficients of 0.80 or higher, and maintains semantic equivalence with the original English version, indicating strong reliability (68–71). The PHQ-9 is a nine-item self-assessment validated instrument that asks participants to rate the frequency of symptoms they've experienced over the past 2 weeks (68–71). The items are based on a Likert scale. The total score ranges from 0 to 27, with higher scores indicating more severe depressive symptoms (PHQ-9 Score: between 0 to 4 = no depression symptoms; 5–9 = mild; 10–14 = moderate; 15–19 = moderately-severe depression; and the severe score of depression from 20 to 27).

### Statistical approach

Statistical analyses were conducted using SPSS version 24. Descriptive statistics were used to summarize the sociodemographic characteristics of caregivers and caretaker. Pearson correlation analyses were performed to examine the relationships between caregiving-related variables, age, gender, and depressive symptoms. Independent samples *t*-tests were conducted to assess differences in depressive symptomatology according to employment status. Finally, a multiple linear regression analysis was carried out to evaluate whether spirituality, age, country, educational attainment, employment status and receives support for caregiving tasks function as protective factors against depressive symptoms. The results of statistical tests were considered statistically significant at *p* < 0.05.

## Results

A total of 1,194 caregivers from Mexico (51.1%), Puerto Rico (32.0%), and Colombia (16.7%) participated in the study. Table 1 presents the demographic characteristics of the study sample, the majority of participants were women (74.3%), reflecting established gender norms in informal caregiving across these cultural contexts. Puerto Rico exhibited the highest proportion of female (84.6%) and filial caregivers (70.2%), suggesting the influence of specific cultural factors. Differences among caregivers in Puerto Rico, Mexico, and Colombia may reflect variations in cultural norms, socioeconomic factors, and healthcare systems. Puerto Rico might offer comparatively greater access to formal support, while Mexico and Colombia could face more challenges due to limited resources and fragmented services.

**Table 1:** Demographic characteristics of the study sample (informal caregivers).

**Characteristics**	**Total**		**Colombia**		**Mexico**		**Puerto Rico**	
	***N***	**%**	***n***	**%**	***n***	**%**	***n***	**%**
Sample	1,194	100	200	16.7	611	51.1	383	32.0
**Gender**								
Female	887	74.3	155	77.5	408	66.8	324	84.6
Male	307	25.7	45	22.5	203	33.2	59	15.4
**Age**								
18–20	18	1.5	3	1.5	15	2.5	0	0
21–24	40	3.4	11	5.5	18	3	11	2.9
25–34	162	13.6	25	12.5	84	13.8	53	13.8
35–44	231	19.4	32	16	127	20.8	72	18.8
45–54	361	30.2	61	30.5	193	31.6	107	27.9
55–59	174	14.6	23	11.5	82	13.4	69	18
60–64	103	8.6	22	11	33	5.4	48	12.5
65–74	80	6.7	16	8	47	7.7	17	4.4
75+	21	1.8	7	3.5	11	1.8	3	0.8
**Marital status**								
Single	384	32.2	71	35.5	206	33.7	107	27.9
Married	407	41.6	63	31.5	275	45.0	159	41.5
Widowed	50	4.2	8	4.0	33	5.4	9	2.3
Divorced	117	9.8	9	4.5	52	8.5	56	14.6
Separated	50	4.2	18	9.0	24	3.9	8	2.1
Live together	83	7.0	28	14.0	18	2.9	37	9.7
Never married	10	0.8	0	0	3	0.5	7	1.8
**Formal knowledge on older adults' caregiver**								
Yes	217	18.2	57	28.5	95	15.5	65	17.0
No	977	81.8	143	71.5	516	84.5	318	83.0
**Remuneration or family economy support for older adult caring**								
No	1,095	91.7	189	94.5	539	88.2	367	95.8
Yes, receives financial support from family	99	8.3	11	5.5	72	11.8	16	4.2
**Family of older adult**								
Yes	1,107	92.7	177	88.5	562	92.0	368	96.1
No	87	7.3	23	11.5	49	8.0	15	3.9
**Relationship with older adult**								
My parents	764	64.0	116	58.0	379	62.0	269	70.2
My son	3	0.3	3	1.5	–		–	
My grandparent	149	12.5	23	11.5	86	14.1	40	10.4
My uncle/aunt	36	3.0	3	1.5	24	3.9	9	2.3
My brother/sister	39	3.3	8	4.0	23	3.8	8	2.1
My spouse	65	5.4	14	7.0	33	5.4	18	4.7
Other	49	7.5	9	4.5	17	2.8	23	6.0
Missing value			24	12.0	49	8.0	16	4.2
**Receives support for caregiving tasks**								
Yes	697	58.4	95	47.5	362	59.2	240	62.7
No	497	41.6	105	52.5	249	40.8	143	37.3
**The caregiver has a job or employment**								
Yes	750	62.8	120	60.0	418	68.4	212	55.4
No	444	37.2	84	42.0	193	31.6	171	44.6
**Work schedule**								
Full time	386	51.4	43	35.8	189	45.2	154	72.6
Part time	139	11.6	21	17.5	92	22.0	26	12.2
Flexible time	210	17.6	52	43.3	130	31.1	28	13.2
Missing value	15	1.3	4	3.3	7	1.6	4	1.8
**Primary income**								
Work	571	47.8	66	33.0	327	53.5	178	46.5
Self-work	172	14.4	40	20.0	100	16.4	32	8.4
Government assistance	73	6.1	17	8.5	29	4.7	27	7.0
Nutritional assistance	31	2.6	2	1.0	1	0.2	28	7.3
Family economic assistance	117	9.8	40	20.0	61	10.0	16	4.2
Other	230	19.3	35	17.5	93	15.2	102	26.6
**Spirituality**								
Yes	1,084	90.8	179	89.5	548	89.7	357	93.2
No	110	9.2	21	10.5	63	10.3	26	6.8

The majority of caregivers (82.6%) were under the age of 60, indicating that caregiving responsibilities are largely assumed by middle-aged adults who may concurrently manage professional and personal obligations. All participants were actively involved in caregiving tasks, supporting at least one (71.5%) or more older adults aged 60 years or older. A majority (74.2%) had provided care for two or more consecutive years. Regarding the setting of care, 61.5% of caregivers provided care in the senior's residence, while 35.2% did so in their own homes. In terms of weekly time investment, 50.2% of caregivers reported providing care for 17 h or more. This level of commitment was highest among caregivers in Colombia (64.8%), followed by Puerto Rico (54.0%) and Mexico (44.0%).

Care recipients were described as requiring assistance most of the time by 44% of caregivers. Despite the intensity of care required, 91.8% of participants reported receiving no financial remuneration for their caregiving activities. Furthermore, 81.8% indicated they lacked formal training in elder care, pointing to a need for structured educational and support programs. Although more than half of caregivers (58.4%) received some assistance with caregiving tasks, nearly 71% reported not having access to psychological support after assuming the caregiving role.

Most caregivers were employed (62.8%), with the highest employment rate observed in Mexico (68.4%). Full-time employment was particularly common in Puerto Rico (72.6%). The primary source of income for caregivers was salaried work (47.8%), though self-employment (14.4%) and government or family assistance were also reported. In terms of educational attainment, caregivers from Puerto Rico reported the highest levels of education (84.6% with some level of post-secondary to doctoral education), followed by those from Mexico (64.4%) and Colombia (39.6%).

Spirituality emerged as an important personal resource, with 90.7% of respondents indicating that it played a significant role in their lives. Marital status data showed that 41.6% of caregivers were married, making it the most common status, although the majority were unmarried—an aspect that may influence the availability of spousal support. Taken together, the findings underscore the gendered nature of caregiving in these contexts, highlighting the prevalence of unpaid, middle-aged, female caregivers who are often immediate family members of the older adults they support.

Caregiver-reported characteristics of the older adults receiving care are summarized in the sample (Table 2). Most older adults were aged 75 or older (64.8%), with Puerto Rico showing the highest proportion in this age group (67.4%). In terms of functional support, 44.6% of care recipients required frequent assistance, particularly in Puerto Rico (49.6%). Occasional help was needed by 36.2%, with Mexico reporting the highest proportion in this category (38.0%). A smaller proportion (19.3%) required only supervision, most commonly in Colombia (21.0%).

**Table 2:** Demographic characteristics of caretaker.

**Characteristics**	**Total**		**Colombia**		**Mexico**		**Puerto Rico**	
	***N***	**%**	***n***	**%**	***n***	**%**	***n***	**%**
**Age**								
60–74	414	34.7	71	35.5	218	35.7	125	32.6
≥75	774	64.8	125	62.5	391	64.0	258	67.4
Missing value	6	0.5	4	2.0	2	0.3	0	0
**Support required by the older adult**								
Only supervision	230	19.3	42	21.0	127	20.8	61	15.9
Sometimes help	432	36.2	68	34.0	232	38.0	132	34.5
Most time help	532	44.6	90	45.0	252	41.2	190	49.6
**Diagnosed with chronic disease**								
Yes	535	56.6	0		289	63.7	246	73.7
No	410	43.3	157	100	165	36.3	88	26.3

Health status information was available for 945 respondents, as this section of the survey was optional. Among them, 56.6% of older adults had at least one chronic condition, with the highest prevalence observed in Puerto Rico (73.7%), followed by Mexico (63.7%). No data were collected in Colombia due to caregiver reluctance to disclose health information, which limits cross-national comparisons for this variable.

Among caregivers who reported receiving some form of support, the majority identified a family member as their primary source (74.1%). This was followed by support from a hired caregiver (25.3%) and, lastly, from their partner (12%; Table 3). It is important to note that participants were allowed to select more than one option, so these categories are not mutually exclusive. Additionally, sixteen caregivers selected the “Other” option, reporting support from various sources such as neighbors, the older adult's extended family, veteran services, nurses, church congregations, home care services, and domestic service personnel.

**Table 3:** Support received by caregivers of older adults.

**Characteristics**	**Category**	**Number (*n* =)**	**Proportion (%)**
Source of support	Family	517	74.1
	Spouse/partner	82	12.0
	Hired person	177	25.3
	Other	16	2.2

The PHQ-9 results revealed a high prevalence of depressive symptoms among caregivers (Table 4). Fatigue was the most frequently reported symptom, with 76.7% indicating low energy. Sleep disturbances (63.3%) and lack of interest or pleasure (46.3%) were also common. Nearly half (49.8%) reported appetite changes, and 13.5% acknowledged thoughts of self-harm—underscoring critical mental health risks in this population.

**Table 4:** Distribution, means and standard deviation of responses to PHQ-9 depression scale.

**Item**	**No**		**Some days**		**More than half the days**		**Nearly every day**		**Mean**	**Std Dev**
	***n***	**%**	***n***	**%**	***n***	**%**	***n***	**%**		
1. Little interest or pleasure in doing things.	641	53.7	319	26.7	153	12.8	81	6.8	0.73	0.929
2. Feeling down, depressed, or hopeless.	549	46.0	396	33.2	162	13.6	87	7.3	0.82	0.925
3. Trouble falling or staying asleep or sleeping too much.	438	36.7	357	29.9	199	16.7	200	16.8	1.13	1.089
4. Feeling tired or having little energy.	278	23.3	446	37.4	271	22.7	199	16.7	1.33	1.010
5. Poor appetite or overeating.	599	50.2	297	24.9	169	14.2	129	10.8	0.86	1.027
6. Feeling bad about yourself or that you are a failure or have let yourself or your family down.	667	55.9	272	22.8	138	11.6	117	9.8	0.75	1.003
7. Trouble concentrating on things, such as reading the newspaper or watching television.	637	53.4	313	26.2	152	12.7	92	7.7	0.75	0.952
8. Moving or speaking so slowly that other people might have noticed? Or the opposite: being so fidgety or restless that you have been moving around a lot more than usual.	790	66.2	229	19.2	110	9.2	65	5.4	0.54	0.872
9. Thoughts that you would be better off dead or of hurting yourself in some way.	1,033	86.5	101	8.5	33	2.8	27	2.3	0.21	6.25

These findings reflect a significant burden of depressive symptomatology, with some caregivers experiencing persistent affective and somatic symptoms. The results of the PHQ-9 questionnaire indicate that, on average, caregivers reported symptoms of significant depression according to the instrument's interpretation. The most frequent symptoms were fatigue, insomnia, and changes in appetite. Suicidal thoughts were reported less frequently (*M* = 0.21), although their presence remains clinically relevant.

**Figure 1:**
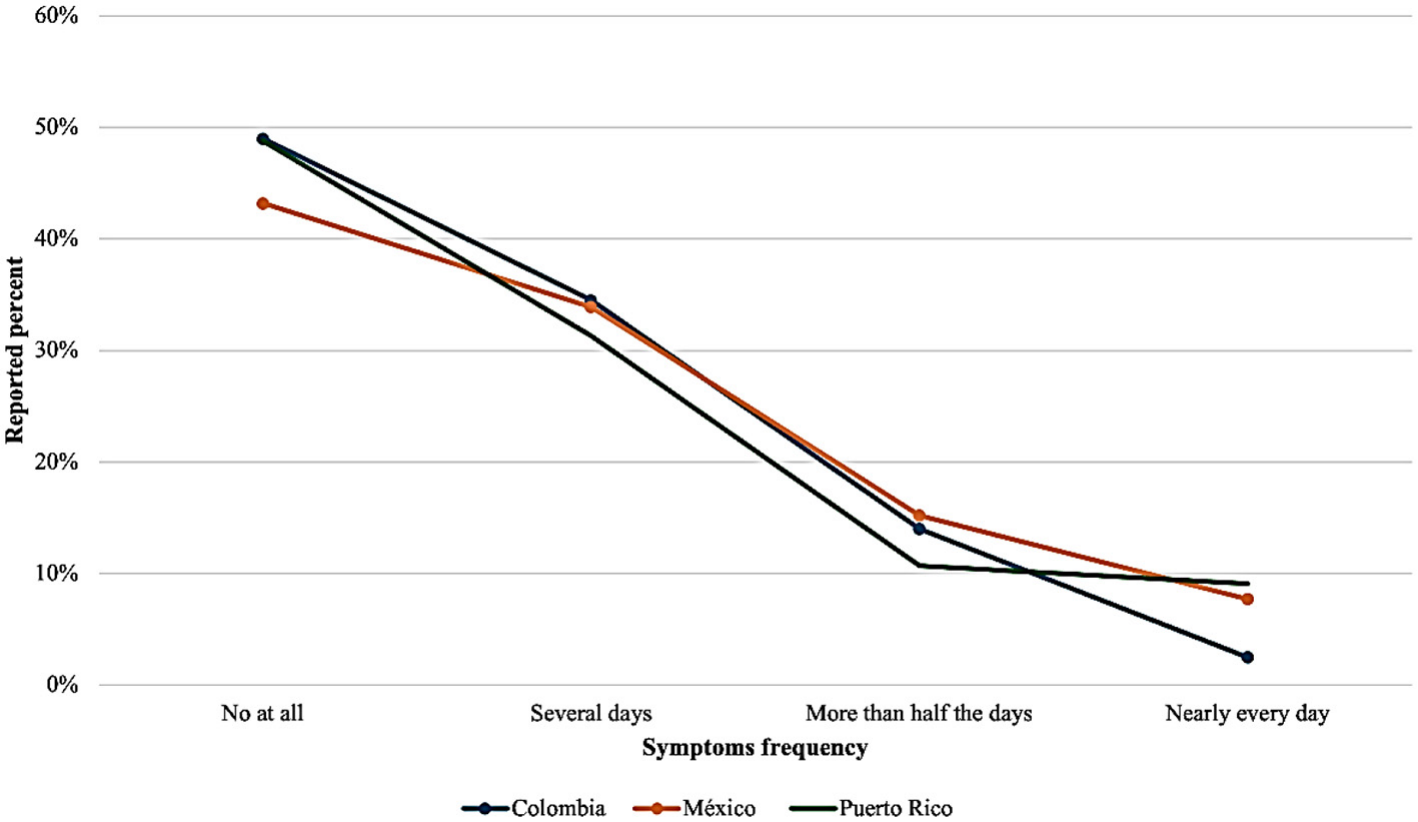
Frequency to PHQ-9 question by country: I do feel discouraged, hopeless, or depressed.

Country-specific patterns in depressive symptoms varied across the sample (Figure 1). In Colombia, 58.0% of the caregivers in our sample reported no loss of interest in daily activities, while 31.5% experienced symptoms on several days. Additionally, 7.5% had persistent symptoms for over half the days, and 3.0% reported a near-daily lack of interest. For suicidal ideation, 82.5% denied thoughts of self-harm, 12.5% reported such thoughts on several days, and 3.0% reported them nearly daily. In Mexico, 53.2% of respondents did not report anhedonia, while 40.7% experienced it several days a week; 6.1% reported it nearly daily. Regarding suicidal ideation, 85.8% denied such thoughts, 9.0% reported them on certain days, and 1.8% nearly daily. Among Puerto Rican caregivers, 52.2% reported no anhedonia, but 27.4% experienced it several days a week. Persistent symptoms were seen in 10.4% of participants, with 9.9% experiencing them almost daily. In this group, 89.8% denied suicidal thoughts, while 5.5% reported them on several days, and 2.6% nearly daily.

The most statistically significant correlations between depressive symptoms and caregiving-related factors are presented in Table 5. All associations reached significance at *p* < 0.05, with the majority surpassing the *p* < 0.01 threshold. Among the variables examined, self-harm thoughts exhibited a moderate positive correlation with depressive symptoms (*r* = 0.511, *p* < 0.01), indicating a meaningful association that reflects elevated psychological distress within this subgroup. Other factors showed weaker, though statistically significant, correlations. Specifically, caring for individuals with special needs (*r* = 0.204) and the amount of time dedicated to caregiving (*r* = 0.191) were both positively associated with depressive symptoms, suggesting a cumulative burden effect. Additional variables, such as being employed (*r* = 0.112), being a daughter (*r* = 0.082), or being a granddaughter (*r* = 0.063), demonstrated small but significant correlations, potentially reflecting the influence of socioeconomicvulnerability and role-based expectations on caregiver mental health.

**Table 5:** Most significant correlations between depression and caregiving factors.

**Factors**	**Correlation coefficient (*r*)**	***p*-Value**
Time spent on caregiving	0.191	< 0.01
Employed	0.112	< 0.01
Special needs caretaker	0.204	< 0.01
Self-harm thoughts	0.511	< 0.01
The caregiver is the daughter	0.082	< 0.05
The caregiver is the granddaughter	0.063	< 0.03

**Figure 2:**
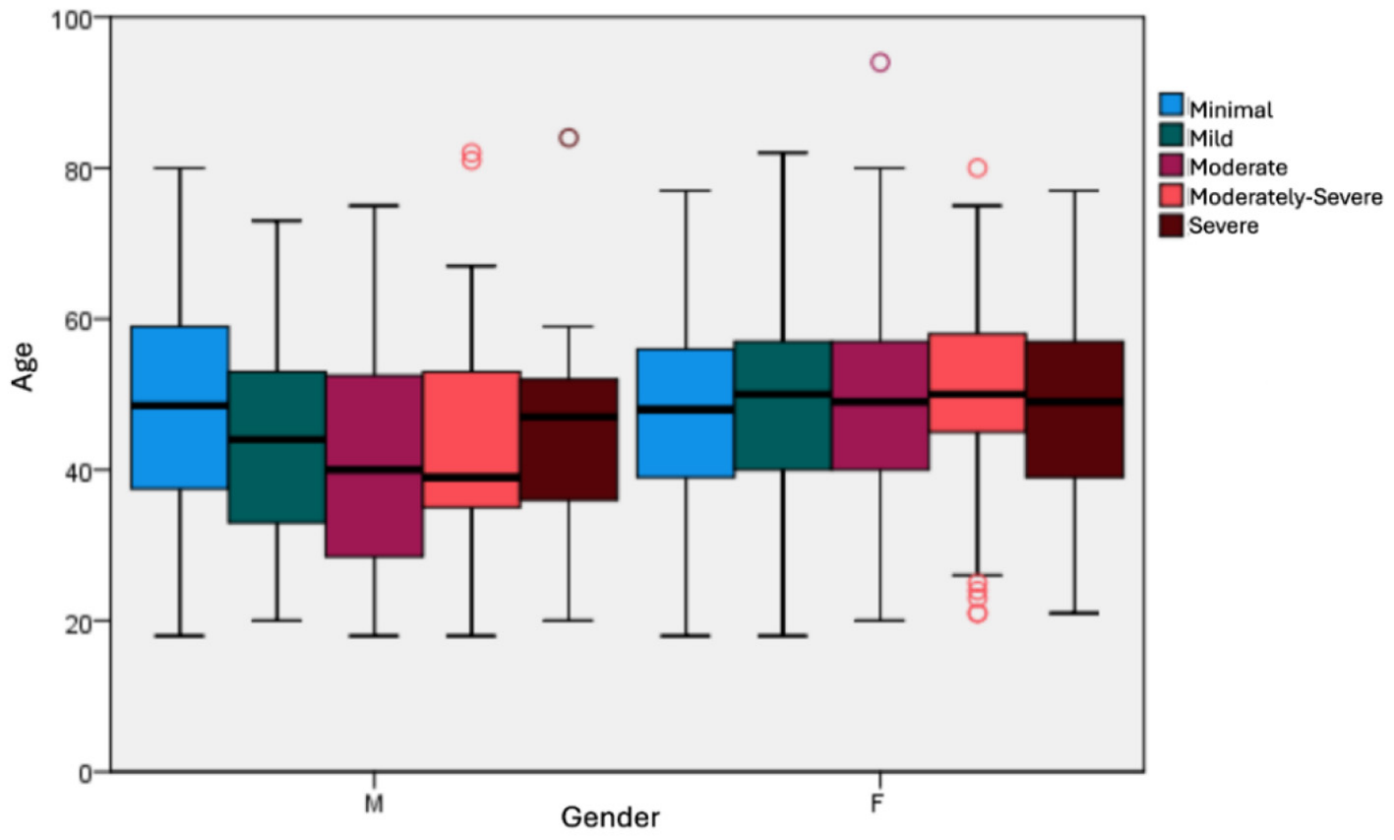
Distribution between depression scoring, age and gender general sample.

In general, females in our sample have higher depression levels than males (Figure 2). The analysis of age-related variations in the sample identifies several notable outliers in depression levels. Overall, the findings indicate that females between the ages of 40 and 60 exhibit significantly higher levels of severe depression than their male counterparts. Additionally, males aged 30–40 appear more likely to experience moderate to moderately severe depressive symptoms. The observation that younger males may be more susceptible than females in this age group underscores the complex emotional landscape of caregiving. In this context, both age and gender may play pivotal roles in determining mental health outcomes.

**Figure 3:**
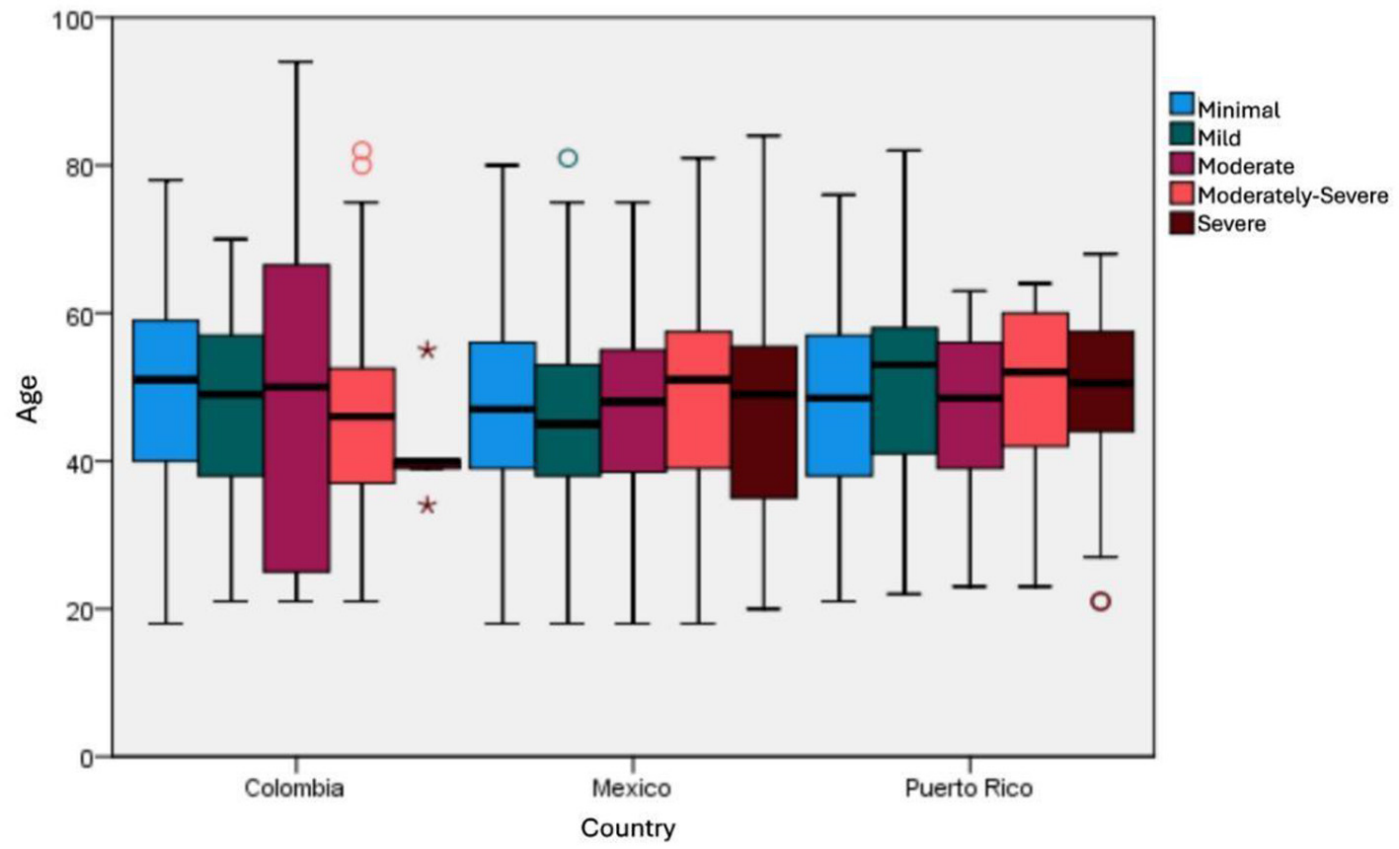
Distribution between depression scoring and age by country. The asterisks (*) and circles (°) denote statistical outliers, with asterisks indicating extreme outliers beyond 3 times the Interquartile Range (IQR) and circles representing mild outliers.

Depression symptoms, analyzed by age and country, have consistently ranged from moderate to high levels (Figure 3). Among the populations studied, caregivers from Mexico and Puerto Rico exhibited the most severe depression levels. In comparison, Colombians reported a broader age range of participants experiencing moderate depression. Overall, across the three countries, individuals aged 40–60 consistently demonstrated the highest levels of depression.

**Figure 4:**
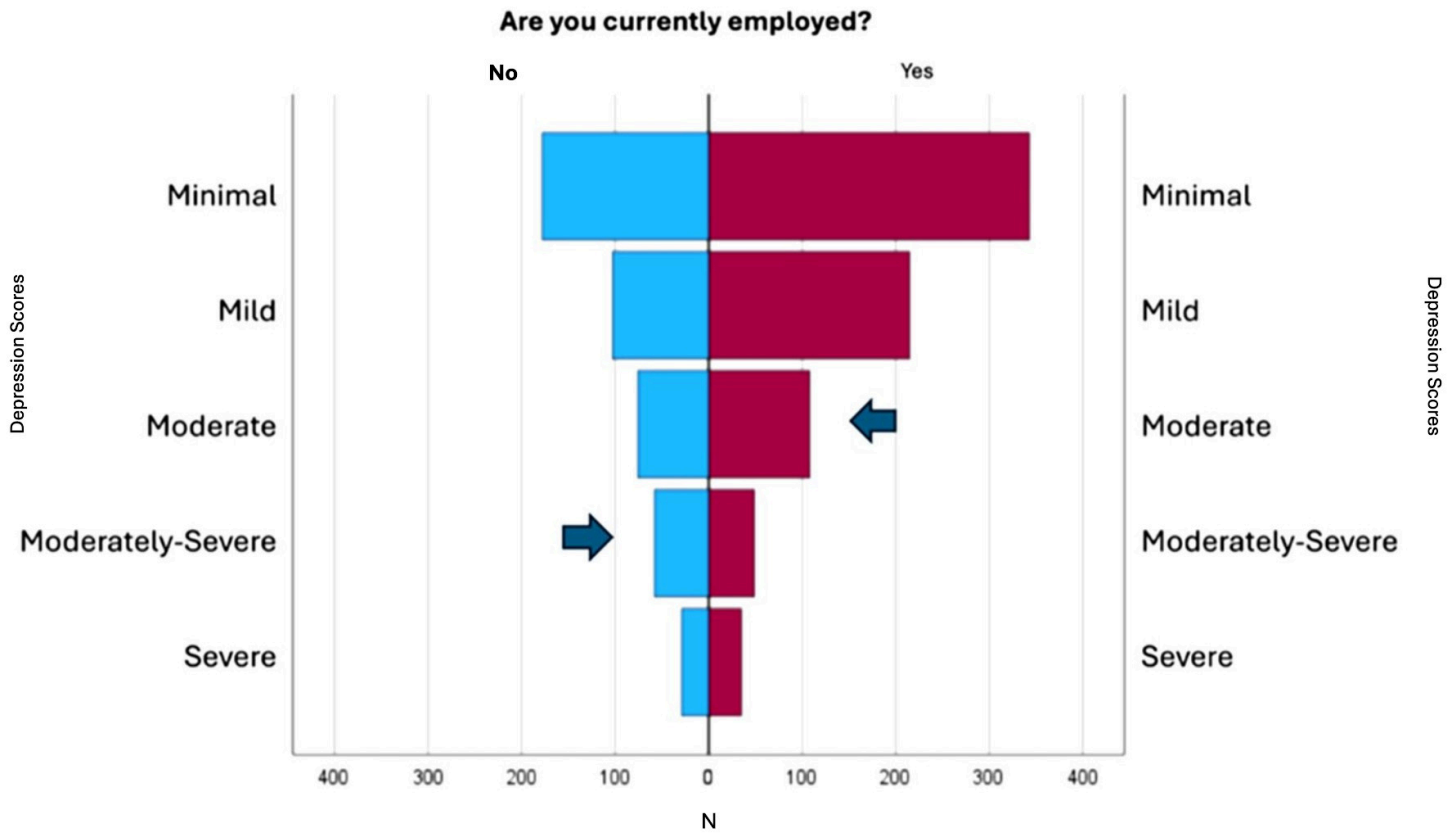
Frequencies between depression scores, caregiving, and employment.

Examining the employment patterns of caregivers across countries provides valuable insight into how work engagement relates to mental health outcomes. Over 68% of the Mexican respondents reported being employed, but only 44% of them worked full-time. In Colombia, 59% of the participants are employed, with 35% working full-time. In contrast, in Puerto Rico, 55% of the participants were employed, with 72% working full-time. Family caregivers frequently encounter difficulties when trying to balance their work and caregiving responsibilities. However, the data collected indicate that employed family caregivers tend to have mild or minimal depression score (Figure 4). In the Pearson correlation analysis between caregiver depression levels and employment status, a statistically significant but weak correlation was identified between those to variables (*p* = < 0.001, *r* = 0.112), suggesting that employment status may play a role in caregiver mental health.

Statistically significant differences were observed in eight of the nine assessed items, indicating a consistent pattern of variation across most symptoms evaluated. Table 6 presents the results of independent samples' *t*-tests assessing depressive symptoms among caregivers based on their employment status. The most notable differences were found in the items “little interest or pleasure in doing things” [*t* (1,192) = −3.64, *p* < 0.001, *d* = −0.218], “feeling down, depressed, or hopeless” [*t* (1,192) = 3.46, *p* < 0.001, *d* = −0.207], and “feeling tired or having little energy” [*t* (1,192) = −3.55, *p* < 0.001, *d* = 0.213]. Mean differences across these items ranged from 0.20 to 0.22 points, with higher scores in the unemployed group. Although the effect sizes (Cohen's *d*) were small (ranging from −0.067 to −0.218), their consistency across symptoms suggests a pattern of increased psychological vulnerability among those without employment. The item “thoughts of being better off dead or self-harm” approached significance (*p* = 0.059), indicating a possible trend toward increased ideation in the unemployed group.

**Table 6:** Differences in depressive symptoms by employment status.

**Item**	***t***	**df**	***p*-Value**	**Cohen's**	**Mean (yes)**	**Mean (no)**
1. Little interest or pleasure in doing things	−3.64	1,192	< 0.001	−0.218	0.65	0.85
2. Feeling down, depressed, or hopeless	−3.46	1,192	< 0.001	−0.207	0.75	0.94
3. Trouble falling or staying asleep or sleeping too much	−3.43	1,192	< 0.001	−0.205	1.05	1.27
4. Feeling tired or having little energy	−3.55	1,192	< 0.001	−0.213	1.25	1.46
5. Poor appetite or overeating	−2.33	1,192	< 0.020	−0.140	0.8	0.95
6. Feeling bad about yourself or that you are a failure or have let yourself or your family down	−2.31	1,192	< 0.021	−0.139	0.7	0.84
7. Trouble concentrating on things, such as reading the newspaper or watching television	−2.33	1,192	< 0.020	−0.132	0.7	0.83
8. Moving or speaking so slowly that other people might have noticed? Or the opposite: being so fidgety or restless that you have been moving around a lot more than usual	−2.72	1,192	< 0.007	−0.142	0.49	0.63
9. Thoughts that you would be better off dead or of hurting yourself in some way	−1.89	1,192	< 0.059	−0.067	0.18	0.25

A multiple linear regression analysis was conducted to evaluate whether spirituality, educational attainment, receiving support for caregiving tasks, employment, country and age function as protective factors against depressive symptoms, while statistically controlling for age and country of origin. The overall model reached statistical significance, *F* (6, 1,187) = 3.215, *p* = 0.004, explaining 1.6% of the variance in depressive symptoms (*R*^2^ = 0.016). Among the predictors, three variables emerged as statistically significant. Educational attainment was inversely associated with depressive symptoms (*B* = −0.528, *p* = 0.022), indicating that higher levels of education may serve as a protective factor. Spirituality (*B* = 1.304, *p* = 0.039) and employment (*B* = 1.452. *p* ≤ 0.01) were positively associated with depressive symptomatology. Conversely, age, country of origin, and receiving support for caregiving tasks were not significant predictors within the model. While the overall explanatory power of the model was modest, the results highlight specific psychosocial variables that may shape the mental health experiences of caregivers (Table 7).

**Table 7:** Linear regression analysis protective factors against depressive symptoms.

**Category**	***B***	**β**	**Depressive symptoms**	
			***t***	***p*-Value**
Constant	4.437		2.944	0.003
Age	0.004	0.035	1.227	0.220
Country	0.222	0.024	0.800	0.424
Education	−0.528	−0.069	−2.301	0.022
Spirituality	1.304	0.060	2.070	0.039
Receives support for caregiving tasks	0.301	0.024	0.819	0.413
Employment	1.452	0.112	3.900	< 0.01

## Discussion

This study investigates the relationship between sociodemographic characteristics and depressive symptoms in informal caregivers across three Hispanic countries linked to the U.S., highlighting key psychosocial predictors and the broader impact of care-related depression in their country of origin. The sample analysis reveals a significant gender imbalance in caregiving, with 74% of caregivers being female, who experience higher levels of depression compared to males. The disproportionate caregiving burden, exacerbated by the fact that 62% of female caregivers are employed and 91.8% receive no financial compensation, reflects entrenched gender roles assigning women primary responsibility for family care.

This research emphasizes the complex relationship between caregiving and depressive symptomatology, providing valuable insights into the psychosocial vulnerabilities that caregivers encounter within their microsystems and familial environments. A high prevalence of depressive symptoms was observed, with fatigue (76.7%), sleep disturbances (63.3%), and feelings of depression or hopelessness (54.4%) emerging as the most reported symptoms. This emotional burden directly impacts caregivers' ability to provide quality care and support to older adults, while also posing long-term health risks for caregivers themselves, especially those offering daily emotional, financial, and practical assistance.

The strong correlation between depressive symptoms and self-harm thoughts (*r* = 0.511, *p* < 0.01) underscores the urgent need for mental health interventions, including systematic screening and crisis support services. The modest but positive associations between caregiving for individuals with special needs and the duration of caregiving suggest that prolonged caregiving responsibilities cumulatively contribute to psychological distress. Notably, depression often remains undetected, despite its potential link to caregiving duration. Hurtado-Vega (51) found that informal caregivers who devote more than 14 h per day to caring for older adults experience higher levels of anxiety, depression, and physical health issues compared to non-caregivers. Consistent with this, our study identified a modest but statistically significant relationship between depression and time spent caregiving. To mitigate the impact of these stressors, concise and effective support strategies, such as cognitive-behavioral group interventions that encourage active participation, may prove beneficial for caregivers (45, 54).

Employment also emerged as a factor associated with elevated depressive symptoms. Although causality cannot be inferred from this correlation, it is plausible that caregivers balancing work responsibilities face compounded psychological distress, exacerbated by economic pressures and the demands of providing care. Additionally, gendered caregiving expectations are evident in our findings, with daughters and granddaughters disproportionately assuming caregiving duties. These results underscore the need for comprehensive, gender-sensitive support systems that mitigate the adverse effects of caregiving and promote caregiver wellbeing. However, it is crucial to approach the relationship between gender, caregiving, and depression with caution. This finding underscores the importance of further research should adopt longitudinal and qualitative approaches to deepen understanding and inform targeted interventions. Some authors exploring gender differences in caregiver profiles have noted that women are often more proactive regarding their health and more inclined to seek healthcare than men (72). These insights can enhance discussions about engagement in activities that provide personal social support.

Our research revealed that more than 58.4% of respondents receive support from various sources, such as family members, romantic partners, professional caregivers, religious community members, or neighbors. The differences in health systems, and caregiver policies across Colombia, Mexico, and Puerto Rico may shape access to formal support services. Variations in healthcare infrastructure and social programs can affect caregivers' ability to obtain training, financial aid, and psychological resources. Liu et al. (18) found that improved family dynamics lead to a significantly better quality of life for caregivers, while increased caregiver overload is associated with a decline in their quality of life. Findings from other studies reveal that caregivers depend on their abilities in acts of selflessness and primarily obtain emotional support from their families and social networks to manage the challenges of caregiving (29).

Nonetheless, our findings indicate that social support is not a significant protective factor against depressive symptoms. Research suggests that a lack of satisfaction with social support and limited assistance contribute to an increased sense of burden among unpaid caregivers of older adults (72). However, factors such as discrimination and language barriers within the community can undermine these crucial connections (46, 73). This is particularly relevant in populations from Mexico and Colombia, where an estimated 1.2 million and 110,500 individuals over the age of 65, respectively, are recognized as speakers of indigenous languages. Although race and ethnicity were not included in our study, some authors suggest that caregiving experiences may vary based on these factors, highlighting the influence of diverse sociocultural backgrounds (15, 36). Further analysis is needed within multiethnic contexts, especially in Mexico and Colombia.

**Figure 5:**
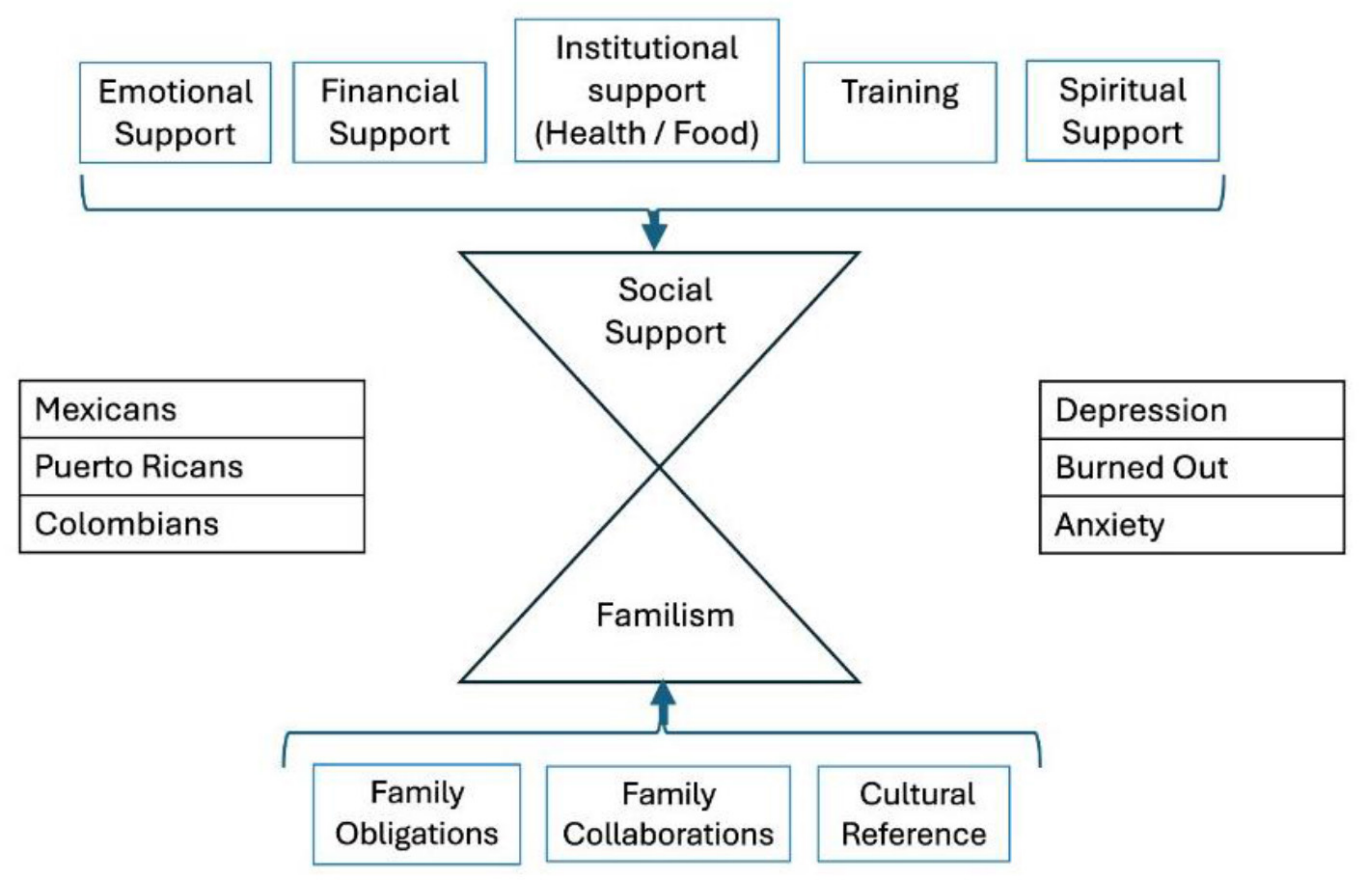
Structural model of race, social support and familism, and social support and familism predicting depression. Adapted from Family matters: cross-cultural differences in familism and caregiving outcomes by Falzarano et al. *J Gerontol B Psychol Sci Soc Sci*. (2022) 5:1275. https://doi.org/10.1093/geronb/gbab160. Copyright © 2021, © The Author(s) 2021. Published by Oxford University Press on behalf of The Gerontological Society of America (19).

Our findings demonstrate that Hispanic family caregivers are more inclined to live with the patient. In this context, some authors compare this to the traditional US family structure and suggest that Hispanics tend to exhibit greater hopefulness, particularly when they are family members (74). In the literature on the United States, some studies suggest that the environments of non-Hispanic Caucasian informal caregivers are marked by lower levels of familism, a lack of reciprocity in their caregiving roles, and generally limited religious beliefs or spirituality. In contrast, our findings, based on data from three Hispanic populations, indicate a different relationship with familism and spirituality. For Hispanic families, the concept of family functionality seems deeply rooted in a commitment to mutual care and support. This commitment entails adapting to changes and collaboratively tackling challenges that arise when a family member requires assistance, highlighting the resilience and strength of familial bonds (34). However, navigating the interplay between various support systems and mental health outcomes within Hispanic communities remains a complex and multifaceted challenge. Framed within the ecological structural-systems model, this study provides insight into the impact of family caregiving on the mesosystem, particularly in how its associated symptoms can affect external environments such as the workplace. Based on our findings, we have adapted Falzarano's conceptualization to underscore the significance of incorporating cultural values in the design of interventions and support systems for family caregivers (Figure 5).

Earlier studies have found that non-working caregivers tended to exhibit more depressive symptoms than their employed counterparts, even after controlling for sociodemographic factors and social networks (75). Our study's *t*-test analysis reveals that unemployed caregivers report higher scores across most PHQ-9 items, with statistically significant differences supported by negative *t*-values and effect sizes (Cohen's *d*). While these differences are not always of large magnitude, they are systematic and reflect a clear trend within the analyzed sample. The most notable differences were found in the items “little interest or pleasure in doing things,” “feeling down, depressed, or hopeless,” and “feeling tired or having little energy.”

The absence of work-related resources may exacerbate psychological distress among unemployed caregivers, potentially hindering their ability to re-enter the labor market and perpetuating a cycle of economic and emotional vulnerability. Notably, although suicidal ideation did not reach conventional statistical significance (*p* = 0.059), the unemployed caregivers warrant attention, as even subclinical elevations in this domain may carry clinical implications. These findings highlight the importance of support programs that enhance caregivers' job stability through initiatives such as work flexibility and access to mental health resources. In this context, financial instability emerges as a significant source of stress that can lead to increased levels of depression and anxiety (57). Caregivers may face limited resources, often forcing them to purchase cheaper, less nutritious food, which negatively impacts their health (51). Financial pressure can harm their nutrition and set the stage for future health problems, including chronic conditions from poor diet and stress.

The study's results from the *t*-test and linear regression analysis provide complementary insights into the factors contributing to depressive symptoms among caregivers. The linear regression suggests that employment, rather than serving as a protective factor, is positively associated with depressive symptoms (*B* = 1.452, *p* < 0.01). The positive coefficient observed in the regression indicates that, within the caregiver population, employment may be linked to an increased psychological burden, potentially due to the dual demand of occupational and caregiving responsibilities. Moreover, the depressive symptoms with the most pronounced differences in the *t*-test, such as fatigue, sleep disturbances, and feelings of worthlessness, are precisely those that can impair workplace functionality. This finding suggests that, while employment provides structural and economic stability, it is not sufficient on its own to prevent depression unless accompanied by favorable working conditions and support resources. Protective factors such as resilience, self-efficacy, purpose, and social support play vital roles in reducing care-related stressors, thereby promoting a holistic quality of life for caregivers (76).

Additionally, the analysis found that higher levels of education may mitigate depressive symptoms, suggesting that greater educational attainment could enhance access to mental health information and foster more effective coping strategies, potentially reducing caregiving-related stress. Conversely, spirituality emerges within the sample as a significant predictor of depressive symptoms. Care practices may have developed from religious observance and a sense of communal responsibility, aimed at alleviating or distributing the burden of caregiving (42, 77). However, the positive coefficient indicates that higher levels of spirituality are associated with an increase in depressive symptoms, an unexpected finding given that previous literature generally identifies spirituality as a protective resource against psychological distress.

One possible interpretation is that, within this caregiver sample, spirituality may be linked to passive coping mechanisms or resignation in the face of caregiving challenges, which could exacerbate depressive symptomatology rather than alleviate it. Additionally, this result may reflect a tendency in which individuals experiencing depressive symptoms rely more heavily on spirituality as a coping strategy, explaining the observed positive correlation in the model. Furthermore, religiosity can affect mental health through the concept of religious scrupulosity; thus, clinicians should exercise caution in order not to over-pathologize cultural ideals or overlook signs of psychopathology (78). The presence of a positive coefficient underscores the need for further exploration of spirituality's role in caregivers' emotional wellbeing, considering the influence of morality and differentiating between resiliencepromoting practices and those potentially associated with feelings of hopelessness or fatalism. Moral orientations are diverse among individuals and are influenced by various contexts and the passage of time (79). This complexity can result in moral distress, a phenomenon that resonates with all parties involved, highlighting the interconnectedness of our experiences. The moral identity assigned to family caregivers often exacerbates the challenges faced by migrant women (80).

These findings underscore the importance of adopting an intersectional lens in understanding caregiver mental health. The data show that most caregivers are middle-aged, Hispanic women providing unpaid care, often without formal training or psychological support. Considering factors such as unemployment, low educational attainment, and gendered caregiving expectations, it becomes clear that these overlapping identities may compound psychological vulnerability. In this context, the regression analysis highlights the uneven impact of key sociodemographic factors. Higher educational attainment was associated with fewer depressive symptoms, while employment and spirituality were both linked to increased symptoms. These contrasting associations suggest that social and cultural resources are not always beneficial; instead, their effects may depend on caregivers' personal experiences, broader social and economic challenges, and the emotional meaning attached to each role.

### Recommendations

From an applied perspective, our findings suggest several targeted intervention strategies to support informal caregivers. First, given the observed association between extended caregiving hours and increased depressive symptoms, it is essential to develop respite care programs specifically directed at caregivers who provide more than 12 h of daily care. These programs should offer flexible, shortterm relief options to reduce psychological distress and prevent burnout. Second, considering the modest but significant relationship between spirituality and caregiver wellbeing, we recommend integrating spiritually oriented components into psychosocial interventions. These could include access to religious social support networks, faith-based counseling, or structured opportunities for spiritual reflection. Including variables such as perceived control and religious social support in intervention design may offer a more nuanced and culturally relevant approach to addressing depressive symptoms in this population. Third, to address the limited access to formal training and mental health resources identified in the caregiver population, public policies should prioritize community-based support networks, affordable psychological services, and education or professional development programs tailored to caregivers' specific needs. These initiatives would not only enhance caregivers' skills but also reduce feelings of isolation and emotional burden.

Finally, it is critical to acknowledge the interplay between micro-systemic family norms and macrosystemic caregiving policies. In the three countries under examination, adult offspring are legally obligated to care for their parents. Therefore, coordinated efforts between government and community institutions should prioritize caregiver subsidies, paid leave, geriatric care training, and in-home support services. Such measures are key to alleviating both the emotional and physical burdens of caregiving, especially for those operating within culturally and structurally reinforced caregiving roles.

## Conclusion

This study reveals consistent predictors of depressive symptoms among informal caregivers in three Spanish-speaking countries: unemployment, lower education, and passive spiritual coping increased distress, while higher education was protective. Formal employment did not reduce symptoms, likely due to the dual burden of work and caregiving. These findings highlight the need for culturally responsive, holistic interventions that address caregivers' spiritual, familial, and economic vulnerabilities. Clinically, this research offers three key insights: First: demographic markers can guide early identification and preventive outreach. Second: the link between employment and higher depressive symptoms underscores the need for workplace mental health support. Third: the connection between spirituality and distress suggests reorienting passive coping into resilience-building strategies. To reduce mental health disparities, future research should employ longitudinal and probability-based designs to explore causal pathways and disparities across sociodemographic variables (gender, socioeconomic status, ethnocultural and migration history). Policymakers and service providers must translate this evidence into action by developing integrated, accessible support systems that include workplace screening, caregiver education, and community partnerships, ultimately improving caregiver wellbeing across diverse cultural settings.

### Limitations and future directions

This cross-sectional study on informal caregivers in three Spanish-speaking countries has inherent design limitations. Convenience sampling risks selection and social desirability biases, hindering generalizability and potentially underrepresenting caregiver distress or marginalizing their challenges. Single-point data collection precludes causal inference. While standardized, our instruments may have constrained nuanced cultural expression, and unequal sample sizes limit cross-country comparability. We also could not deeply explore sociocultural factors like ethnicity and stigma influencing symptom reporting.

Despite these, our findings offer valuable insights for Hispanic communities. Future research should employ mixed-methods and probabilistic sampling for enhanced representativeness and deeper understanding of lived experiences. Subsequent work must investigate how high-demand jobs, limited social support, and migratory mourning exacerbate caregiver strain, particularly for those facing cultural displacement, loneliness, and social isolation. Investigating the impact of economic hardship and food insecurity on caregiver wellbeing and care quality during crises such as forced displacement or migration is crucial. There is a notable lack of understanding regarding how migration affects informal caregivers, highlighting the need for culturally sensitive assessments and interventions. This need is further aligned with the requirement for preventive mental health strategies, particularly within formal employment contexts, as occupational stress in conjunction with caregiving responsibilities can exacerbate depressive symptoms. Such symptoms adversely affect not only occupational performance but also personal wellbeing. Additionally, further analysis is needed to understand the vulnerabilities of culturally displaced caregivers and age-related factors that may intensify depression.

## Data Availability

All data generated or analyzed in this study are include in the article. Further inquiries can be directed to the corresponding authors.
